# CNN and SVM-Based Models for the Detection of Heart Failure Using Electrocardiogram Signals

**DOI:** 10.3390/s22239190

**Published:** 2022-11-26

**Authors:** Jad Botros, Farah Mourad-Chehade, David Laplanche

**Affiliations:** 1 Computer Science and Digital Society Laboratory (LIST3N), Université de Technologie de Troyes, 10300 Troyes, France; 2 Pôle Santé Publique, Hôpitaux Champagne Sud (HCS), 10000 Troyes, France

**Keywords:** deep learning, machine learning, binary classification, convolutional neural network, support vector machine, heart failure, electrocardiogram

## Abstract

Heart failure (HF) is a serious condition in which the heart fails to supply the body with enough oxygen and nutrients to function normally. Early and accurate detection of heart failure is critical for impeding disease progression. An electrocardiogram (ECG) is a test that records the rhythm and electrical activity of the heart and is used to detect HF. It is used to look for irregularities in the heart’s rhythm or electrical conduction, as well as a history of heart attacks, ischemia, and other conditions that may initiate HF. However, sometimes, it becomes difficult and time-consuming to interpret the ECG signal, even for a cardiac expert. This paper proposes two models to automatically detect HF from ECG signals: the first one introduces a Convolutional Neural Network (CNN), while the second one suggests an extension of it by integrating a Support Vector Machine (SVM) layer for the classification at the end of the network. The proposed models provide a more accurate automatic HF detection using 2-s ECG fragments. Both models are smaller than previously proposed models in the literature when the architecture is taken into account, reducing both training time and memory consumption. The MIT-BIH and the BIDMC databases are used for training and testing the adopted models. The experimental results demonstrate the effectiveness of the proposed framework by achieving an accuracy, sensitivity, and specificity of over 99% with blindfold cross-validation. The models proposed in this study can provide doctors with reliable references and can be used in portable devices to enable the real-time monitoring of patients.

## 1. Introduction

Heart failure (HF) is an abnormal condition in which the heart is unable to pump blood at a sufficient rate to meet systemic metabolic requirements [1]. It is a clinical syndrome caused by structural and functional myocardium defects that impair ventricular filling or blood ejection. Reduced left ventricular myocardial function is the most common cause of HF [2]. Increased hemodynamic overload, ischemia-related dysfunction, and a variety of other conditions are among the major pathogenic mechanisms that lead to HF [3]. It is a leading cause of morbidity and mortality, as well as high healthcare costs, imposing a significant burden on both the patient and society. HF primarily affects the elderly, and incidence and prevalence rise sharply with age in those over 60. The most commonly cited prevalence estimate for the adult population is 1–3% and 5–9% for those 65 and older [4]. HF has been designated as a global pandemic because it affects approximately 26 million people worldwide [5].

The electrocardiogram (ECG) is a non-invasive diagnostic modality that records the rhythm and electrical activity of the heart [6]. The ECG is extremely sensitive for detecting HF [6,7] and plays an essential role in predictive monitoring tools [8]. It is used to look for irregularities in the heart’s rhythm or electrical conduction, as well as a history of heart attacks, ischemia, and other conditions that can lead to HF [3,9]. The accurate and rapid interpretation of an extended duration ECG signal monitoring of a subject places a significant burden on cardiologists. The availability of an intelligent, self-learning, and prognostic ECG analysis system can help cardiologists quickly interpret an ECG signal. This type of system allows for the early and accurate detection of heart abnormalities [10].

Artificial intelligence is a branch of computer science that aims to make computers smarter. Learning is a fundamental requirement for any intelligent behavior. Most researchers today agree that intelligence cannot exist without learning [11]. Various machine learning and deep learning methods have been proposed in the literature to address the problem of HF detection. To detect HF, the authors propose a Bayesian classifier [12], Classification And Regression Trees (CART) [13], support vector machines [14], and random forests [15]. Heart Rate Variability (HRV) measures extracted from HRV signals, or ECG signals are used as inputs to these models. The proposed machine learning models achieve varying degrees of accuracy, sensitivity, and specificity. To confront the issues with machine learning, various authors used a variety of deep learning algorithms to address the same concern. Deep Neural Networks (DNN) [16], Long Short-Term Memory networks (LSTM) [17], Convolutional Neural Networks (CNN) [18,19,20,21,22], and the Inception module in conjunction with LSTM [20,23] are all designed to detect HF. As inputs, these models take ECG and RR interval signals. When tested, these deep learning models achieve high evaluation metrics.

This article proposes a 7-layer deep CNN and its extension by incorporating an SVM as the classification layer for HF automatic detection with a short-time ECG signal record. The automatic detection of HF using both models is more accurate than previously proposed models in the literature. Their architecture is also smaller, which reduces training time and memory consumption. These techniques rely on a 1-D CNN. CNNs are hierarchical neural networks that mimic the human visual system and have been shown to be effective in image classification, localization, and detection tasks, among other things. Furthermore, they have been widely used in classification tasks for time series analysis. Arrhythmia detection and multivariate diagnostic measurement modeling are two successful examples. The main advantage of using a CNN over traditional machine learning methods is that it detects important features automatically without the need for human intervention. The proposed architecture is smaller than the currently available architectures in the literature, with seven layers, and thus consumes less memory and computation time. The proposed models require no engineered features and only minimal pre-processing of ECG signals. The models are trained and tested using a balanced dataset created from the MIT-BIH and BIDMC databases, which are obtained from ambulatory Holter and ECG recorders, respectively, achieving an accuracy, sensitivity, and specificity of over 99% with blindfold cross-validation.

The rest of the article is organized as follows. Section 2 presents the related work. Section 3 describes the databases and the proposed methods. Section 4 presents and discusses the obtained results. Finally, Section 5 provides the conclusion.

## 2. Related Works

To address the HF diagnosis problem, many machine learning and deep learning techniques have been proposed in the literature. In his study, Asyali investigates the discrimination power of nine commonly used long-term HRV measures. He then uses a Bayesian classifier to categorize ECG signals as healthy or HF based on the standard deviation of NN intervals, abbreviated as SDNN. The obtained accuracy is 93.24%, with sensitivity and specificity of 81.82% and 98.1%, respectively [12]. Melillo et al. distinguish between healthy and heart failure patients using a combination of HRV measures fed into a CART model. The classification accuracy, sensitivity, and specificity were 96.36%, 89.74%, and 100%, respectively [13]. Liu et al. describe a method for detecting HF using three different combinations of HRV measures and an SVM classifier. In terms of accuracy, sensitivity, and specificity, the model is absolutely perfect when cross-validation is used instead of testing on unseen data [14]. Masetic et al. achieve the same result through feature extraction and classification. The features are extracted using the auto-regressive burg method and then fed into a random forest classifier, which achieves 100% accuracy, sensitivity, and specificity [15].

Data pre-processing is an important step in machine learning techniques because the quality of the data, and thus the information that can be extracted from it, has a direct influence on the network’s learning ability. Feature selection is another crucial step in machine learning. The selection process can be labor-intensive and time-consuming. In order to avoid the pitfalls of traditional machine learning, deep learning models are used to optimize the performance of a Computer-Aided Diagnosis (CAD) system. Using a deep learning model based on sparse auto-encoders (SAE), Chen et al. detect HF using RR interval segments. The SAE is used to extract some features, which are then classified by a DNN. The results show that the accuracy is 72.44%, the sensitivity is 50.93%, and the specificity is 80.93% [16]. Wang et al. present a deep learning method for classifying ECG signals as healthy or HF using LSTM. The network is fed a 500-point RR interval segment. The accuracy of the model is 84.91%, its sensitivity is 75.49%, and its specificity is 90.06% [17]. Acharya et al. propose an 11-layer CNN to classify ECG signals as healthy or HF. The accuracy is 98.97%, the sensitivity is 98.87%, and the specificity is 99.01% [18]. Wang et al. detect HF using LSTM and an inception module, in which they replace one convolutional block with an LSTM network. The network achieves 86.42% accuracy, 74.91% sensitivity, and 91.21% specificity for a 500-point RR interval segment input [23]. Padmavathi et al. present an 11-layer CNN method for detecting HF. The model has an accuracy rate of 80.10%, a sensitivity rate of 81%, and a specificity rate of 79.30% [19]. Lih et al. develop a CNN-LSTM model with 16 layers (one of which is an LSTM with 10 units) for ECG signal classification. This one is 98.5% accurate, 99.3% sensitive, and 97.89% specific [20]. Zhang et al. refine the DenseNet model and apply it to HF detection using 2-s ECG fragments obtained after noise filtering, R-peak detection, and segmentation of long-term ECG signals. The model achieves 94.97% accuracy, 89.38% sensitivity, and 99.50% specificity [21]. Porumb et al. use a 1-D CNN for feature extraction and the Multi-Layer Perceptron (MLP) for classifying raw ECG heartbeats. This hybrid model attains an accuracy of 97.8%, a sensitivity of 96.3%, and a specificity of 98.6% in classifying healthy and HF patients [22]. Table 1 summarizes the evaluation metrics of the different methods found in the literature.

## 3. Materials and Methods

### 3.1. Databases Description

**Figure 1:**
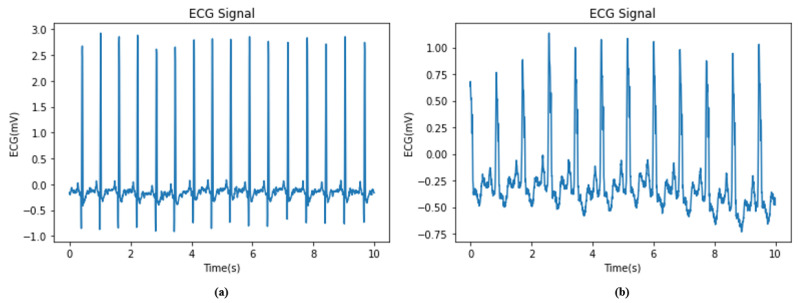
The raw ECG signals. (**a**) A healthy subject. (**b**) A HF patient.

The experimental data for this retrospective study are gathered from Physionet and divided into two parts. The first part comes from the MIT-BIH database and is for Normal Sinus Rhythm (NSR) [24]. It is made up of the ECG signals of 18 healthy individuals, including 5 men between the ages of 26 and 45 and 13 women between the ages of 20 and 50. The second part comes from the BIDMC database for Congestive Heart Failure (CHF) [25]. It is made up of the ECG signals of 15 heart failure patients, including 11 men aged 22 to 71 years old and 4 women aged 54 to 63 years old. The signals are approximately 20 h long and are collected using ambulatory Holter and ECG recorders. Each record includes two channels of the ECG, with a sampling rate of 128 Hz for the MIT-BIH NSR database and 250 Hz for the BIDMC CHF database. Figure 1 shows the raw ECG signals of a healthy subject and a HF patient, and Table 2 summarizes the characteristics of each database.

### 3.2. Pre-Processing

At first, the MIT-BIH NSR recordings are up-sampled to 250 Hz to guarantee that all ECG signals are sampled at the same frequency. Then, a non-repeated division of the long-term sequence is conducted. Every 2 s is selected as a fragment. The last fragment was removed if it was not longer than 2 s. This leads to approximately 648,000 segments of 500 points in length in the healthy group and 540,000 segments of 500 points in length in the HF group. After that, Z-score normalization is applied, as shown in Equation (1), to regularize the segments such that the mean is 0 and the standard deviation is 1. This ensures that all segments contribute equally to the analysis.

(1) $$z_{i}=\left(x_{i}-\mu_{i}\right)/\sigma_{i},$$

where $z_{i}$ is the normalized signal, $x_{i}$ is the original *i*-th 500-point segment, $\mu_{i}$ is its corresponding mean, and $\sigma_{i}$ is its corresponding standard deviation.

With an unequal representation of the two classes, the obtained dataset is unbalanced (55% healthy and 45% HF). To avoid the problem of data imbalance, a balanced dataset is created by taking an equal number of ECG segments from the MIT-BIH NSR and the BIDMC CHF databases. The resulting dataset is generated by randomly selecting an equal number of segments from each of the 15 HF patients and 15 out of 18 healthy subjects, randomly selected as well, to guarantee an equal contribution of each class to the analysis. More fragments could be taken, but this would result in longer computation times and higher memory consumption. We let $y_{i}$ be the label of the segment $z_{i}$; that is, $y_{i}=1$ for HF subjects and $y_{i}=0$ otherwise. The obtained dataset ${\left(z_{i},y_{i}\right)}_{i}$ will be used to train and test the model in the following steps.

### 3.3. The Methods

This paper proposes two deep classifiers for detecting HF. They take a 2-second 500-point normalized segment $z_{i}$ as input and return ${\hat{y}}_{i}=1$ if the subject is HF and 0 otherwise. The first classifier is a CNN model, while the second is an extension of the first by adding an SVM as the classifier.

#### 3.3.1. The CNN Model

**Figure 2:**
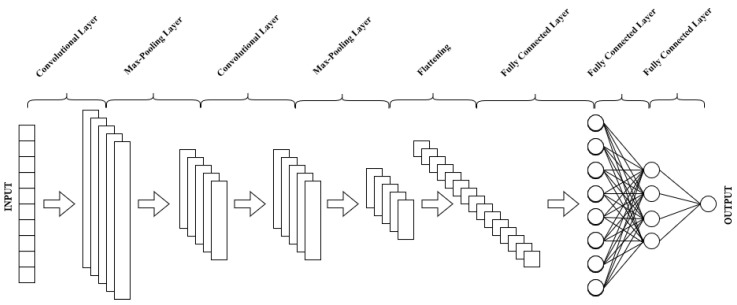
General structure of the proposed model.

The first classifier proposed in this paper is a seven-layer CNN. The model is built up of two convolutional layers, two max-pooling layers, and three fully connected layers. The main advantage of using a CNN over traditional machine learning techniques is that it can detect important features automatically without the need for human intervention. Its lack of reliance on pre-processing reduces the need for human effort while expanding its capabilities. Indeed, the convolutional layers, which consist of a fixed number of filters, are used to automatically extract the feature maps. Convolutional layers convolve their vector signal with their different filters according to Equation (2) in which $b={\left(b\left(n\right)\right)}_{n}$ is the result of the convolution of the signal $a={\left(a\left(k\right)\right)}_{k}$ by the kernel (·), *n* and *k* are positional indexes, and *N* is the size of the vector a. The max-pooling layer is used to reduce the network’s dimensionality by taking the maximum value in a specific filter region. The fully connected layer, which is typically placed before the output layer, aggregates data from the final feature map and generates the final classification. Figure 2 shows the general structure of the proposed network.

(2) $$b\left(n\right)=\sum_{k=0}^{N-1}a\left(k\right)\cdot f\left(n-k\right),$$

The first layer receives a 2-s ECG segment with 500 points as input. It is a convolutional layer with five 1×13 filters applied with a stride of 1, resulting in five feature maps generated by convolving the various filters with the 500-point input ECG signal according to Equation (2). The second layer is a max-pooling layer with a pool size of two and a stride of four. This layer reduces the dimensions of the feature maps by convolving a 1×2 filter with each of the previously generated feature maps. As a result, both the number of parameters to learn and the amount of processing in the network are reduced. Consequently, the model is more robust to changes in feature position in the input. Following that, another convolutional layer of ten filters of size 1×9 each is applied with a stride of one. This set of filters is used to extract higher-level features from dimensionally reduced feature maps. The fourth layer is a max-pooling layer with the same properties and tasks as the first. After that are three fully connected layers with 40, 20, and 2 units, respectively. The first layer’s input is simply a flattened version of the previous layer’s output. With the exception of the final layer, which employs the softmax activation function, all layers employ the Leaky ReLU activation function.

Glorot uniform initialization is used to initialize the model weights, and backpropagation with a batch size of 64 is used to update them. The model is constructed over thirty epochs. Consider ${\hat{q}}_{i}$ as the predicted probability that segment *i* is HF, as obtained at the network’s output. The binary cross-entropy function is used to calculate the model’s loss in the binary classification problem, as shown in the following equation:

$$L\left(q\right)=\frac{-1}{M}\Sigma_{i\in I_{T}}y_{i}.\log\left({\hat{q}}_{i}\right)+\left(1-y_{i}\right).\log\left(1-{\hat{q}}_{i}\right),$$

where $I_{T}$ is the set of indices of the segments used for training the model, *M* is the total number of these segments; that is, *M* is the cardinal of $I_{T}$, and *q* is the index of the epoch. The cross-entropy computes a score that represents the mean difference between the actual and the predicted values. The score is to be minimized, where the 0 value is a perfect cross-entropy.

The parameter space of this model is tuned using grid search and trial and error. Grid search, a tuning technique, is used to find the best hyperparameter values. It is a process that searches exhaustively through a manually specified subset of the targeted algorithm’s hyperparameter space. Grid Search is used to tune the batch size and number of epochs in this study, while trial and error is used to choose the filter sizes and strides.

#### 3.3.2. The CNN-SVM Model

**Figure 3:**
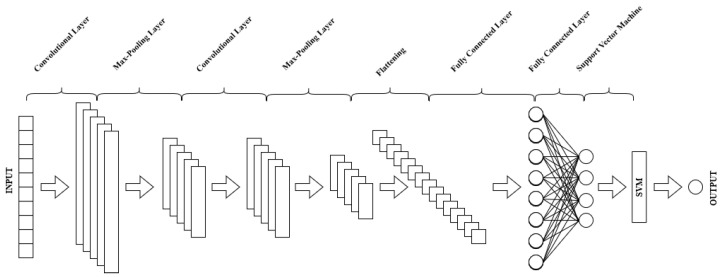
General structure of the proposed hybrid model.

This section presents an extension of the proposed network. Instead of keeping the last layer of the CNN network, which is responsible for the final classification, we replace it with an SVM classifier. This is called ensemble learning, which refers to algorithms that combine the predictions from two or more models. Ensemble learning has been applied in the medical field and shows promising results in QRS complex detection and classification, as well as arrhythmia detection [26,27]. In other words, a pruned CNN network is kept to extract features, and then the classification is performed based on SVM. Therefore, a CNN-SVM hybrid model is proposed next for the classification of ECG signals from the two databases. The proposed system combines the best characteristics of SVM and CNN classifiers. The CNN is trained and uses self-learning to extract the feature maps that are fed to the SVM for binary classification [28]. CNN operates in a similar manner to humans and is capable of learning invariant local features very well. It is capable of extracting the most distinguishing information from raw signals. The support vector machine is a classification algorithm that learns to differentiate between binary labels of given data. It is an algorithm that learns to label objects by example. In essence, an SVM is a mathematical algorithm that maximizes a specific mathematical function with respect to a given set of data [29]. It finds the separating hyperplane that divides data by maximizing the margin of a dataset. The margin is defined as the shortest distance between two data points separated by a hyperplane. SVM is a linear method that has been extended to include non-linear problems by projecting the data to a higher dimensional space. This method has been particularly successful due to the ease with which it handles high-dimensional data and the interpretability of its linear models [30]. SVM has been found to be good for binary classification but poor for noisy data. The shallow architecture of SVM makes learning deep features difficult. The hybrid CNN-SVM model is proposed in this work, in which a non-linear SVM is used as a binary classifier and replaces CNN’s softmax layer. The proposed hybrid CNN-SVM model’s architecture is described in Figure 3.

The optimal hyperparameter values are tuned using grid search, which is a tuning technique. It is an optimization method that tests a series of parameters and compares the performances in order to come up with the optimal parameterization. For each parameter, a set of test values is determined. The Grid Search simply crosses each of these hypotheses and creates a model for each combination of parameters. In this study, different combinations of C and gamma are tested. C is the error term penalty parameter, and it represents the degree of correct classification that the algorithm must achieve, whereas gamma specifies how much curvature is desired in a decision boundary.

## 4. Results and Discussion

Two CNN models are proposed in this article to classify healthy subjects and patients with HF. In determining the performance of the proposed models, combined approaches are used. A common strategy is to divide the entire dataset into three independent subsets: a training set for training the models, a validation set for optimizing and fine-tuning the hyperparameters, and a testing set for evaluating the models’ performance. Due to the limited number of data available, and in order to present each class in all subsets in the appropriate proportions, 15 out of 18 healthy subjects are chosen randomly in addition to the 15 HF patients. Three out of each group are then randomly selected for the validation set and similarly for the testing set. The remaining 18 subjects are used for training. Another effective approach is stratified cross-validation, which divides the dataset into an equal number of folds with an equal percentage of each class in each fold. One fold is used for testing each time, and the remaining folds are used to train the models. A modified 10-fold stratified cross-validation is used in this study. The 2-second segments of data collected from nine patients from each group are used to train the models in each iteration. Similarly, data from three different patients in each group are used to validate the models, while the remaining data (from three additional patients in each group) are used to test the models. The procedure is carried out ten times. The average of each evaluation metric is then computed. When evaluating classifier performance, three terms should be introduced: accuracy, sensitivity, and specificity. Accuracy is defined as the number of classifications a model correctly predicts divided by the total number of predictions made, as shown in Equation (3). Sensitivity describes how well the classifier recognizes positive samples and is defined by Equation (4), in which TP represents the number of true positive samples and FN represents the number of false negative samples. It specifies the number of people who have congestive heart failure and have test results that are positive. Specificity describes how well the classifier recognizes negative samples and is defined by Equation (5), in which TN represents the number of true negative samples and FP represents the number of false positive samples. It defines the number of people who are healthy and have negative test results.

(3) $$Accuracy=\frac{TP+TN}{TP+FN+TN+FP}$$

(4) $$Sensitivity=\frac{TP}{TP+FN}$$

(5) $$Specificity=\frac{TN}{TN+FP}$$

Table 3 and Table 4 summarize the aforementioned measures for the executed ten folds for the CNN and CNN-SVM models, respectively. As can be seen, the presented CNN model achieves an average accuracy of 99.73%, with an average sensitivity of 99.58%, and an average specificity of 99.83% for the created dataset, while the hybrid CNN-SVM model achieves an average accuracy of 99.26%, with an average sensitivity of 99.37%, and an average specificity of 99.11%, for the same dataset.

When the tenth fold, with which the performance of the two models drops, is ignored and the standard deviation of the three evaluation metrics is calculated, the standard deviation of the CNN-SVM model is smaller. The standard deviation of the accuracy is 0.55 vs. 0.47, the sensitivity is 0.42 vs. 0.37, and the specificity is 0.99 vs. 0.85, respectively, for the two models. A low standard deviation indicates that the data are tightly clustered around the mean, implying that the data are more reliable. Furthermore, when the average of the nine-fold evaluation metrics is recomputed, the ensemble model clearly outperforms the CNN model alone with 99.60% vs. 99.62% average accuracies, 99.71% vs. 99.74% average sensitivities, and equal average specificities. One possible explanation of the model’s behavior of this tenth fold might be the noise and the differences between the training and the testing data in that particular fold.

The selected number of epochs allows the loss function to converge and reach a stable minimum. Python was used to write the code, which was then implemented in Google Colab.

To demonstrate the performances of the proposed models, a comparison is made between the obtained results and those of other HF detection methods using the same databases. As a result, five existing methods are considered. The authors of [13] investigated the discrimination power of various HRV measures and selected three combinations of these measures to form the input to a CART classifier. The method in [18] used an 11-layer CNN to classify 2-s ECG fragments, minimally pre-processed, into healthy and HF. Prior to beats segmentation, the authors of [19] up-sampled the ECG signals to a common frequency, applied empirical-mode decomposition to filter these signals, and performed R-peak detection. The beats were fed into an 11-layer CNN also for classification. The authors in [21] filtered the ECG signals to remove the noise that may affect them and detected the R-peak before segmenting the signals into 2-s fragments. The method in [22] segmented the raw ECG signals into beats that were fed to a CNN classifier. Table 5 compares the test evaluation metrics of the different methods from the literature to the proposed models. The results of the method in [18] are shown in Table 5, with an unbalanced dataset in the upper row. When balanced data are used, accuracy falls to 94.40%, as do sensitivity and specificity, which fall from 98.87% to 94.68% and from 99.01% to 94.12%, respectively, as shown in the lower row. The results demonstrate that the proposed model outperforms existing methods. The methods of [13,21] achieve a higher specificity but at the expense of sensitivity and accuracy, which are lower than those obtained with the proposed models. When compared to traditional machine learning methods, the use of deep learning in the presented method, along with efficient training and a suitable architecture, ensures high performance. Deep learning capabilities, in fact, allow for more reliable signal abstraction in high-dimensional space without the need for human intervention. When compared to the deep model’s architectures in [18,19,21,22], the proposed model is smaller and faster when training time is taken into account.

The findings of this study must be viewed in light of some limitations. Data are collected from the ECG signals of a small group of participants, so the results may not be generalizable to all patients and signals. This has an impact on the model’s training and testing phases, as well as its generalizability to previously unseen data and data acquired using different devices. The ECG signals were obtained primarily from ambulatory Holters and ECG recorders. As a result, the sample is unlikely to represent the entire spectrum of signals, particularly those acquired using different devices. Another limitation could be the lack of patient follow-up, which makes it impossible to detect patient status degradation.

## 5. Conclusions

An automated ECG classification approach is developed in this article, and an extension of it is proposed for detecting congestive heart failure. The developed model is a fully automatic seven-layer CNN that requires no feature extraction or selection stages and requires no pre-processing of ECG signals. The proposed extension replaces the model classification layer with an SVM. In this extension, the features are extracted automatically using the CNN and are fed to the SVM for HF detection. ECG recordings of healthy subjects are obtained from the MIT BIH NSR database, while ECG recordings of heart failure patients are obtained from the BIDMC CHF database. The two databases are freely accessible on the internet. A dataset is created with 120,000 healthy ECG segments and 120,000 ECG segments from heart failure patients to avoid data imbalance issues. The robustness of the proposed networks is confirmed by ten-fold cross-validation. The presented CNN classifier, which achieved high metrics, plays an important role in the detection and classification of ECG signals, as evidenced by its accuracy of 99.31%, sensitivity of 99.50% reflecting the true positive rate, and specificity of 99.11%, which represents the true negative rate when the dataset is balanced. The extended version also achieved high evaluation metrics, as evidenced by its accuracy of 99.17%, sensitivity of 99.74%, and specificity of 98.61% when the dataset is balanced. Future work could include the fusion of various medical data, such as time series, punctual measurements, and clinical notes, to ensure efficient monitoring of a subject’s state over time and to provide a more precise classification with stratification of patients into different levels of heart failure. The classification problem would then be defined as a multi-class hybrid classification, combining data of various types.

## Figures and Tables

**Table 1:** The evaluation metrics of the different methods found in the literature.

Method	Classifier	Accuracy	Sensitivity	Specificity
[12]	Bayesian Classifier	93.24	81.82	98.10
[13]	CART	96.36%	89.74%	100%
[14]	SVM	100%	100%	100%
[15]	RF	100%	100%	100%
[16]	DNN	72.44%	50.93%	80.93%
[17]	LSTM (100 units)	84.91%	75.49%	90.06%
[18]	CNN (11 layers)	98.97%	98.87%	99.01%
[23]	Inception Module LSTM (5 units)	86.42%	74.91%	91.21%
[19]	CNN (11 layers)	80.10%	81%	79.30%
[20]	CNN-LSTM (16 layers, 10 units)	98.5%	99.3%	97.89%
[21]	Densenet	94.97%	89.38%	99.50%
[22]	CNN-MLP	97.80%	96.30%	98.60%

**Table 2:** The characteristics of the databases.

Database	Number of Signals	Properties
MIT-BIH NSR	18 Long-Term ECG Signals	5 men between 26 and 45 13 women between 20 and 50 fs = 128 Hz
BIDMC CHF	15 Long-Term ECG Signals	11 men between 22 and 71 4 women between 54 and 63 fs = 250 Hz

**Table 3:** Ten-fold evaluation metrics of the CNN model (Balanced dataset).

Fold	Accuracy (%)	Sensitivity (%)	Specificity (%)
1	99.88	99.92	99.84
2	99.89	99.95	99.82
3	99.32	98.73	99.90
4	99.31	99.31	99.30
5	98.32	99.72	96.92
6	99.98	99.99	99.97
7	99.91	99.82	100
8	99.97	99.97	99.98
9	99.90	99.98	99.81
10	96.64	97.66	95.62
Average (%)	99.31	99.50	99.11

**Table 4:** Ten-fold evaluation metrics of the hybrid CNN-SVM model (Balanced dataset).

Fold	Accuracy (%)	Sensitivity (%)	Specificity (%)
1	99.85	99.87	99.84
2	99.90	99.97	99.82
3	99.40	98.90	99.90
4	99.14	99.35	98.94
5	98.60	99.80	97.40
6	99.97	99.99	99.95
7	99.90	99.80	99.99
8	99.97	99.95	99.99
9	99.81	99.98	99.63
10	95.23	99.82	90.63
Average (%)	99.17	99.74	98.61

**Table 5:** Evaluation metrics of the different methods found in the literature.

Method	Accuracy (%)	Sensitivity (%)	Specificity (%)
[12]	96.36	89.74	**100**
[18] unbalanced	98.97	98.87	90.01
[18] balanced	94.40	94.68	94.12
[19]	80.10	81	79.30
[21]	94.97	89.38	99.50
[22]	97.80	96.30	98.60
Proposed CNN Model	**99.31**	99.50	99.11
Proposed CNN-SVM Model	99.17	**99.74**	98.61

## Data Availability

MIT-BIH Normal Sinus Rhythm ECG signals database at https://www.physionet.org/content/nsrdb/1.0.0/ (accessed on 30 October 2022) and BIDMC Congesitve Heart Failure ECG signals database at https://physionet.org/content/chfdb/1.0.0/ (accessed on 30 October 2022).
